# Letters from the Honorary Secretaries of King's Fund and the Treasurer of St. George's

**Published:** 1912-05-18

**Authors:** 


### ST. GEORGE'S HOSPITAL AND KING EDWARD'S FUND.

### Letters from the Honorary Secretaries of King's Fund and the Treasurer of St. George's.

Under the heading "Hospital and Hospital Funds," we last week called attention to the leading article in *The Times* of May 2 which contained remarkable inaccuracies and was a departure from the well-established practice of *The Times* by failing to be judicial and impartial throughout. The Honorary Secretaries of the King's Hospital Fund in *The Times* of the 10th inst. correct the remarkable inaccuracies which occurred in *The Times* article, and put the facts clearly. In republishing this statement we are confident that hospital opinion will be practically unanimous in thanking Sir Savile Crossley and Mr. John G. Griffiths for the facts which they have prepared and published.

### THE RELATION BETWEEN THE KING'S FUND AND ST. GEORGE'S HOSPITAL.

TO THE EDITOR OF "THE TIMES."

Sir,—May we by your courtesy have the opportunity of enlightening your readers on the subject of the relation between King Edward's Hospital Fund and St. George's Hospital, dealt with in the leading article in *The Times* of the 2nd inst.? The conclusions reached in the article appear to be based on false premises, and the following statement may assist in their correction.

1. The article states that the difficulty arose out of representations made to the Fund by Mr. Stephen Coleridge and by Mr. Garrity, the secretary of the Metropolitan Radical Federation.

This is an error. The difficulty arose out of an important change made by St. George's in the method of dealing, in their accounts, with certain payments to the medical school in respect of the cost of laboratories used by hospital and school. The Fund, having previously accepted the old method, had intimated to St. George's beforehand that, if the change were made, it could not accept the new one without investigation. More than a year afterwards Mr. Coleridge and the Federation attacked both the Fund and St. George's on a different ground—namely, that all payments to medical schools, even for hospital work, were contrary to the finding of Sir Edward Fry's Committee. The Fund had no difficulty in showing that the report of that committee expressly authorised payments for specific work done in respect of the treatment of patients.

2. The article states that the Fund then appointed a committee whose members possessed almost every kind of knowledge except that germane to the matters at issue.

The sub-committee appointed to conduct the investigation, of which St. George's had notice a year earlier, consisted of four of the most experienced members of the Distribution Committee—one a past president and one a Fellow of the Royal College of Physicians, and two past hon. secretaries of the Fund, both of whom had had a large acquaintance with hospital work and finance, while one had been chairman of a hospital and hon. secretary of a Medical Research Fund. It is difficult to know on what grounds *The Times* denies to these gentlemen the capacity to form a judgment on a question relating to hospital administration.

3. The article says that in the present year a grant was withheld from St. George's, not upon the previous ground that there had been a contribution to medical education, but on different grounds. It goes on to explain these other grounds by saying that St. George's had in the meantime obtained discretionary powers from donors sufficient to cover the alleged contribution to the school, and that the Fund therefore changed its ground and charged St. George's with spending more upon the work in question than the average at other hospitals.

This allegation in itself contains three separate inaccuracies:—

(*i.*) No question of refusing a grant to St. George's arose at the last distribution, because St. George's withdrew its application on learning the previous decision.

(*ii.*) The discretionary fund at St. George's had existed for many years, and the whole difficulty arose because the hospital ceased to utilise the discretionary powers given by the donors to that fund for the purpose of enabling them to pay money to the medical school.

(*iii.*) There was no change of ground on the part of the King's Fund. The comparison between the cost of the work in question at St. George's and the average at other hospitals was part of the evidence upon which the Fund based its conclusion that the apportionment of the cost of the laboratories as between hospital and school was faulty and involved an indirect contribution to the school.

4. The article accuses the Fund of laying down an average cost per bed for laboratory work which may not be exceeded by any hospital.

The Fund has never done any such thing. Its investigation showed that the cost to the hospital of the laboratory work at St. George's was about 180 per cent. above the average at other hospitals, and was largely in excess of the previous cost as shown in their accounts; and the earlier course of the inquiry having thrown doubts upon the method by which the apportionment of the expenses in question between hospital and school was arrived at, the Fund came to the conclusion that part of this excess was due to faulty apportionment. But the amended apportionment which the Fund requested St. George's to make left the cost of their laboratory work still nearly 50 per cent. above the average, a margin which the Fund considered ample for any differences due to variations either in the quality of the work or in the economy of management.

It must be borne in mind that King Edward's Hospital Fund is governed by the statement made in 1897 by its founder, King Edward VII., then Prince of Wales, that "there was no intention of devoting any part of the Fund towards the support of the medical laboratories."

We are, Sir, your obedient servants,

Savile Crossley,
John G. Griffiths,
Honorary Secretaries.

King Edward's Hospital Fund for London,
7 Walbrook, E.C., May 8.

### LETTER FROM THE TREASURER OF ST. GEORGE'S.

The Treasurer of St. George's Hospital, in *The Times* of the 14th inst., publishes a long letter occupying more than a column dealing with the above communication from the Honorary Secretaries of the King's Fund. The Treasurer does not directly meet any of the points so clearly set forth in the letter from the King's Fund. Much space is taken up by details which, so far as the ordinary reader can follow them, do not seem material to the issues.

The case which the Treasurer presents on behalf of St. George's Hospital appears to be that the King's Fund has shifted its ground on three occasions. Mr. West writes : "The original charge was that money was diverted for medical education. This is altered to a question of comparative cost, and now we are told we have indirectly contributed to the school." Does not the Treasurer here merely beg the question, seeing that each of the three details he enumerates directly relates to the main point at issue—namely, that the St. George's Hospital authorities should enter in the Uniform System of Accounts their Discretionary Fund, as they did formerly, and that they will now and henceforth follow the course adopted by all other hospitals with medical schools which receive a grant from the King's Fund? With the exception of one short paragraph, which seems to us merely to reveal that he is not conscious of the extent to which he has in reality begged the question, we give the latter part of the Treasurer's letter in full :—

"There are two matters of great importance involved in this question. The first is that all expenditure upon patients in a hospital, whether in money or in kind, must be taken into account when trying to arrive at comparative costs or in comparing the expenditure of one hospital with another, and this is not now done. The second is—Are the King's Fund to be the final judges of a proper expenditure upon the development of one or another department of a hospital's work? And, if so, why is one hospital selected and the rule not made universal? I would respectfully suggest that an independent committee be appointed to consider the whole question of the keeping of hospital accounts, as I am confident that anyone with a full knowledge of the facts must admit that the present method does not show the real comparative cost as between the various hospitals, which factor has a large bearing upon the question of the grants made by King Edward's Fund."

Will Mr. West kindly elaborate this latter proposal of his? The King's Fund and all hospitals which follow completely the Uniform System do in fact keep accurate hospital accounts, and so do show the real comparative cost as between the various hospitals. What more can hospital accounts accomplish, and what useful purpose could the proposed independent committee fulfil?

